# Serum Sirtuin 1, 3 and 6 Levels in Acute Myocardial Infarction Patients

**DOI:** 10.5935/abc.20190114

**Published:** 2019-07

**Authors:** Emrullah Kızıltunç, Arzu Kösem, Can Özkan, Burcu Uğurlu Ilgın, Harun Kundi, Mustafa Çetin, Ender Ornek

**Affiliations:** 1 TC Saglik Bakanligi Ankara Numune Egitim ve Arastirma Hastanesi - Cardiology, Ankara - Turkey; 2 TC Saglik Bakanligi Ankara Numune Egitim ve Arastirma Hastanesi - Medical Biochemistry, Ankara - Turkey; 3 TC Saglık Bakanlıgı Gazi Mustafa Kemal Devlet Hastanesi - Cardiology, Ankara - Turkey; 4 Beth Israel Deaconess Medical Center - Cardiology, Boston, Massachusetts - USA

**Keywords:** Sirtuins/drug effects, Atherosclerosis, Lipid Metabolism, Endothelium/dysfunction, Cardiomegaly, Cellular Senescence, Carcinogenesis

## Abstract

**Background:**

Sirtuins may act in many cellular processes like apoptosis, DNA repair and lipid/glucose metabolism. Experimental studies suggested some sirtuin types may have protective effects against endothelial dysfunction, atherosclerosis, cardiac hypertrophy and reperfusion injury. Data about sirtuins in acute myocardial infarction (AMI) patients are scarce.

**Objectives:**

To investigate temporal changes of serum sirtuin 1,3 and 6 levels in AMI patients; to compare the serum sirtuin 1,3 and 6 levels between AMI patients and control subjects; and to investigate the association of serum sirtuin 1,3 and 6 levels with prognostic markers of AMI.

**Methods:**

Forty patients with AMI and 40 patients with normal coronary arteries were included. Left ventricular ejection fraction (LVEF), serum proBNP, CRP, sirtuin1, sirtuin 3 and sirtuin 6 levels were processed. Peak troponin T levels, GRACE score, first day / second day sirtuin levels were recorded of AMI patients. A p value < 0.05 was considered statistically significant.

**Results:**

Serum sirtuin 1,3 and 6 levels in AMI patients were similar to those in normal coronary patients. No temporal change in serum sirtuin 1,3 and 6 levels were found in AMI course. No correlation was evident between the sirtuin levels and the following parameters: proBNP, CRP, peak troponin and LVEF. Baseline sirtuin 1 and 6 levels were positively correlated with reperfusion duration. Baseline sirtuin 3 levels were negatively correlated with GRACE score.

**Conclusion:**

Serum sirtuin 1,3 and 6 levels in AMI patients were similar to those in normal coronary patients. This study does not represent evidence of the possible protective effects of sirtuin1, 3 and 6 in AMI patients.

## Introduction

Sirtuins are NAD (nicotinamide adenine dinucleotide) dependent enzymes which consist of seven members called Sirt 1-7.^1^ The best known function of sirtuins is deacetylation, but they can also function as mono ADP ribosyltransferase, lipoamidase, demalonylase and desuccinylase.^2,3^ Sirtuins are involved in several biological processes like apoptosis, cellular survival, DNA repair/cellular aging and lipid/glucose metabolism.^4^ The information about the functions of sirtuins in carcinogenesis, aging and inflammation is also increasing.^4,5^ Additionally, there exist evidences that circulating sirtuin levels may associate with frailty, reduction of body fat mass or diabetes mellitus.^6-8^ Contemporary knowledge about the functions of sirtuins in the cardiovascular system in health and disease states is also evolving. Recent experimental studies have demonstrated the possible role of sirtuins in various cardiovascular pathologies like cardiac hypertrophy, heart failure, endothelial dysfunction and atherosclerosis.^9,10^

Survival of the acute myocardial infarction (AMI) patients have significantly improved with the advanced catheter-based therapies and increased availability of coronary care units, but this resulted increase in heart failure population.^11^ Infarct size reduction is crucial to decrease the probability of clinical heart failure and to improve prognosis in AMI patients. Early reperfusion and reduction of reperfusion injury are basic management approaches to reduce the infarct size. However, there exist many variables ascertaining infarct size and only a little is known about the underlying sophisticated molecular mechanisms. Inflammation, thrombogenicity, and oxidative stress effect infarct size and prognosis.^12-14^ Experimental studies revealed that sirtuin 1, 3 and 6 have beneficial effects against atherosclerosis, dyslipidemia, oxidative stress, endothelial dysfunction and inflammation. In addition, sirtuin 1 and 3 may activate cardioprotective pathways in the setting of AMI.^9^ Therefore, serum sirtuin levels may associate with reduced myocardial infarct size and good prognostic markers in AMI patients. To the best of our knowledge, there is no data about the association between serum sirtuin 1,3 and 6 levels and prognostic markers of AMI patients in English-language literature. The goal of this pilot study is to investigate temporal changes of serum sirtuin 1,3 and 6 levels in AMI patients, to determine if there is any difference in serum sirtuin 1,3 and 6 levels between AMI patients and control subjects and to investigate the association between serum sirtuin 1,3 and 6 levels and prognostic markers of AMI patients like peak serum troponin levels, serum pro-BNP levels, GRACE score and post-MI LVEF.

## Methods

The study protocol was approved by the local ethical committee and informed consent forms were obtained from all participants. We enrolled patients with acute ST-segment elevational MI (STEMI) and patients with normal coronary arteries in this cross-sectional study between June 2017 and November 2017. Patients with STEMI were diagnosed according to the third universal definition of myocardial infarction document.^15^ A primary percutaneous coronary intervention was performed and all of the STEMI patients received guideline-mediated medical therapy. GRACE risk score^16^ and TIMI risk score^17^ of the AMI patients were calculated. The duration between symptom onset to reperfusion, location of the infarction in electrocardiogram and presence of pre-infarction angina pectoris were recorded. Serum lipid levels, renal and hepatic function test results, complete blood count results, fasting blood glucose levels, and peak troponin T levels were also recorded. Serum C reactive protein levels and pro BNP levels were processed on the first day of ischemic insult in AMI patients. Venous samples were obtained at the admittance, 24th hour and 48th hour of the infarction for sirtuin 1,3 and 6 analysis to see whether there was any temporal change in serum sirtuin levels after myocardial infarction. Serum lipid levels, renal and hepatic function test results, complete blood count results and fasting blood glucose levels were obtained from local laboratory records and venous samples were collected for C reactive protein, pro BNP and sirtuin level analysis after the coronary angiography in normal coronary artery patients. Transthoracic echocardiography was performed and the LVEF, end diastolic diameter, septal and posterior wall thickness were recorded in all patients. All of the samples for sirtuin level analysis were centrifuged at 4000 rpm for 10 minutes, serums were separated and were frozen at -80 degrees Celsius. All sirtuin 1,3 and 6 levels were processed simultaneously with human sirtuin ELISA kits (YL Biotech, Shanghai, PRC).

Excluded from the study were as follows: The patients with a past history of MI, stable coronary artery disease, peripheral arterial disease, heart failure and any valvular heart disease, the patients with malignancy, active infection, any chronic inflammatory disease, and any chronic renal or hepatic disease.

### Statistical analysis

SPSS 18.0 software for Windows (SPSS Inc. Chicago, IL) was used for analysis of data. For continuous variables, the normality of distribution was tested using Kolmogorov-Smirnov test. The results were presented as the mean ± standard deviation for variables with normal distribution and as median (interquartile range 25-75) for variables with abnormal distribution. The statistical comparisons of continuous variables were performed using independent samples t-test or Mann-Whitney U test regarding the distribution pattern. The comparisons of categorical variables were performed using Chi-square test or Fisher’s exact test. While investigating the association between sirtuins and prognostic markers of AMI, the correlation coefficients and their significance were calculated using the Spearman test. In AMI patients, serial serum sirtuin 1,3 and 6 levels were processed at the admittance, first day and the second day of the infarction and the Friedman test was conducted to test whether there was a significant temporal change in serum sirtuin levels in AMI patients. A p value < 0.05 was considered statistically significant.

## Results

**Figure 1:**
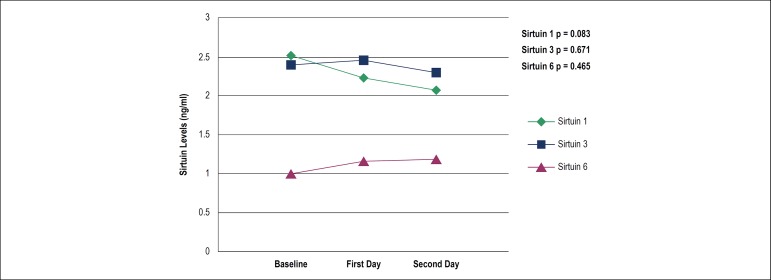
Baseline, first day and second day median serum sirtuin levels of the acute myocardial infarction patients. Temporal changes of serum sirtuin 1,3 and 6 were statistically insignificant in acute myocardial infarction course.

Forty consecutive STEMI patients and 40 consecutive control patients with normal coronary arteries were included into the study. Baseline clinical and laboratory characteristics of the patients were shown in table 1. White blood cell count, neutrophil count, serum creatinine, CRP, proBNP values and left ventricle mass index were significantly higher in the AMI group than in the control group. The control group was composed of more female patients and less smoking patients. The mean platelet count and the mean left ventricular ejection fraction were significantly higher in the control group than in the AMI group. There was no significant temporal change in serum sirtuin levels in AMI patients (Figure 1).

**Table 1:** Clinical and laboratory characteristics of the study patients

	Control patients n = 40	AMI patients n = 40	p Value
Age, y	59 ± 9	57 ± 14	0.481
Sex, Male, n(%)	23(57.5)	37(92.5)	< 0.001
Hypertension, n(%)	20(50)	14(35)	0.175
Smoking, n(%)	8(20)	27(67.5)	< 0.001
Diabetes Mellitus, n(%)	16(40)	11(27.5)	0.237
Family History for CAD, n(%)	4(10)	10(25)	0.077
Hyperlipidemia, n(%)	3(7.5)	5(12.5)	0.456
Fasting blood glucose, mmol/L	5.8(5.3-6.7)	5.9(5.4-8.2)	0.164
Creatinine, µmol/L	75 ± 16	84 ± 14	0.006
Total cholesterol, mmol/L	4.9 ± 1.0	4.8 ± 0.9	0.675
HDL, mmol/L	1.1 ± 0.3	1.0 ± 0.2	0.101
LDL, mmol/L	2.7 ± 0.9	2.9 ± 0.8	0.392
Triglyceride, mmol/L	4.3 ± 2.2	3.9 ± 2.2	0.385
Hemoglobin, gr/dl	14.6 ± 1.9	14.7 ± 1.8	0.904
Platelet count, *10^3^	279 ± 70	229 ± 52	0.001
WBC, *10^3^	8.5 ± 1.9	11.6 ± 3.1	< 0.001
Neutrophil count, *10^3^	5.1 ± 1.4	8.8 ± 3.1	< 0.001
Lymphocyte count,*10^3^	2.6 ± 0.9	2.0 ± 0.9	0.001
Monocyte count, *10^3^	0.8 ± 0.9	0.7 ± 0.3	0.581
CRP, nmol/L	28.5(9.5-57.1)	85.7(38.1-180.9)	< 0.001
proBNP,ng/L	48.8(24.2-113.0)	500.9(282.0-1309.0)	< 0.001
**Sirtuins,ng/ml**			
Sirtuin 1 basal	2.74(2.30-3.64)	2.53(2.06-3.21)	0.192
Sirtuin 1 first day	NA	2.24(1.89-2.89)	
Sirtuin 1 second day	NA	2.08(1.55-3.18)	
Sirtuin 3 basal	2.62(2.16-3.34)	2.40(1.29-3.29)	0.204
Sirtuin 3 first day	NA	2.46(1.37-2.97)	
Sirtuin 3 second day	NA	2.30(1.36-3.55)	
Sirtuin 6 basal	1.13(0.89-2.25)	1.00(0.78-1.37)	0.172
Sirtuin 6 first day	NA	1.16(0.87-1.56)	
Sirtuin 6 second day	NA	1.19(0.85-1.80)	
**EF**, %	64 ± 2	47 ± 8	< 0.001
LVMI, gr/m^2^	87.3 ± 14.6	94.4 ± 16.3	0.044

Continuous variables were presented as mean ± standard deviation or medial (interquartile range 25-75) AMI: acute myocardial infarction; BNP: brain natriuretic peptide; CAD: coronary artery disease; CRP: c reactive protein; EF: ejection fraction; HDL: high density lipoprotein; LDL: low density lipoprotein; LVMI: left ventricle mass index. WBC: white blood cell count.

Clinical and laboratory features of AMI patients were shown in table 2. Fifteen patients experienced pre-infarction angina pectoris before the ischemic insult. Median peak troponin level was 41.6 ng/L in patients without pre-infarction angina and 28.2 ng/L in patients with pre-infarction angina (p = 0.202). Baseline serum sirtuin 1, 3 and 6 levels were similar in the patients with and without pre-infarction angina. No correlation was evident between baseline serum sirtuin levels and following parameters: CRP levels, peak troponin T levels, proBNP levels and LVEF. Baseline sirtuin 1 and 6 levels were positively correlated with reperfusion duration. On the other hand, baseline sirtuin 3 levels were significantly negatively correlated with GRACE score (Table 3).

**Table 2:** Clinical and laboratory features of AMI patients (n = 40)

Presence of Pre-infarction Angina, n(%)	15(37.5)
Time to Perfusion (minutes)	225(120-300)
**GRACE Score **	
In hospital mortality	126(104-149)
6. month mortality	101(77-124)
In hospital MI/mortality	188(151-209)
6. month MI/mortality	148(121-167)
**Killip Class**	
1	38(95)
2	1(2.5)
3	1(2.5)
TIMI Risk Score	2(1-4)
**Troponin T Levels(ng/L)**	
Baseline	4.67(1.00-34.27)
First day	28.50(7.08-58.70)
Second day	14.11(6.46-39.95)
Peak	30.18(10.53-63.40)
**MI Location n(%)**	
Anterior	17(42.5)
Inferior	22(55)
Lateral	1(2.5)
**Infarct Related Artery n(%)**	
LAD	18(45)
Cx	3(7.5)
RCA	19(47.5)
Contrast Induced Nephropathy n(%)	4(10)

*AMI: acute myocardial infarction; Cx: left circumflex artery; LAD: left anterior descending artery; MI: myocardial infarction; RCA: right coronary artery; TIMI: thrombolysis in myocardial infarction.*

**Table 3:** Correlation analysis of prognostic variables of AMI patients with sirtuins

	Baseline Sirtuin 1	Baseline Sirtuin 3	Baseline Sirtuin 6
TIMI Score (Spearman’s Rho/p)	0.109/0.508	-0.093/0.574	0.015/0.930
**GRACE Score (Spearman’s Rho/ p)**			
In hospital mortality	-0.003/0.983	-0.478/0.002	-0.115/0.486
6. month mortality	-0.001/0.997	-0.351/0.028	-0.137/0.406
In hospital MI/mortality	0.045/0.785	-0.509/0.001	0.041/0.805
6. month MI/mortality	0.021/0.901	-0.501/0.001	-0.016/0.922
proBNP, (Spearman’s Rho/ p)	0.294/0.073	-0.137/0.412	0.108/0.517
Peak Troponin T (Spearman’s Rho/ p)	-0.107/0.518	-0.259/0.111	0.012/0.942
Time to Perfusion (Spearman’s Rho/ p)	0.331/0.037	-0.249/0.121	0.312/0.050
CRP, (Spearman’s Rho/ p)	0.312/0.053	-0.029/0.862	0.357/0.026
EF, (Spearman’s Rho/ p)	-0.009/0.956	0.150/0.356	-0.132/0.419

AMI: acute myocardial infarction; BNP: brain natriuretic peptide; CRP: c reactive protein; EF: ejection fraction; TIMI: thrombolysis in myocardial infarction.

## Discussion

In this pilot study, we investigated serum sirtuin 1, 3 and 6 levels in AMI patients and patients with normal coronary arteries. We found that median serum sirtuin levels were similar in AMI patients and control patients. We observed that serum sirtuin levels did not show a significant temporal change in AMI course. There was no correlation between serum sirtuin levels and prognostic markers of AMI patients like peak troponin, proBNP, CRP and ejection fraction. Serum sirtuin 1 and 6 levels were positively correlated with reperfusion duration. In addition, we found a significant negative correlation between serum sirtuin 3 levels and GRACE score.

Atherosclerotic cardiovascular disease is the leading cause of death all over the world.^18^ Primary and secondary prevention attempts are evolving to decrease the global burden of this devastating disorder.^19^ Atherosclerosis pathogenesis is complicated and researches on the molecular basis of atherosclerosis proceed all over the world. In this context, this study evaluated the implication of serum sirtuin 1, 3 and 6 levels in AMI patients. Preliminary data about the sirtuins in cardiovascular diseases were obtained from experimental studies. It was demonstrated that sirtuin 1 exhibits antiatherogenic properties via acting on endothelium by endothelial nitric oxide synthase activation and reducing macrophage foam cell formation by nuclear factor kB inhibition.^20,21^ Another study showed that sirtuin 1 decrease serum LDL cholesterol levels.^22^ It was found that sirtuin 1 have antithrombotic effect in addition to the other atheroprotective effects that were mentioned above.^23^ Oxidative stress plays an important role in the pathogenesis of atherosclerosis and previous studies reported the antioxidant role of sirtuin 3.^24,25^ Sirtuin 6 has anti-inflammatory and LDL lowering features.^26,27^ Human data about sirtuins and cardiovascular disorders are limited. Gorenne et al.^28^ reported that sirtuin 1 expression is reduced in human carotid atherosclerotic plaque.^28^ Breitenstein et al.^29^ found that peripheral monocyte sirtuin 1 expression was lower in coronary artery disease patients compared to healthy subjects.^29^ Judging by our results, this study may arise a debate about the protective functions of circulating sirtuin 1, 3 and 6 in cardiovascular disorders. The first issue is about the association between atherosclerosis and sirtuins. In our study population, median serum sirtuin 1, 3 and 6 levels did not significantly differ between the groups. According to our knowledge, this study is the first one comparing ‘serum’ sirtuin levels between normal coronary artery patients and AMI patients. There exist several studies evaluating sirtuin 1 levels in human atherosclerosis using different methodological manners. Breitenstein et al.^29^ measured sirtuin 1 mRNA levels in peripheral monocytes and Gorenne et al.^28^ measured sirtuin 1 mRNA and protein expression amount in human carotid endarterectomy materials.^28^ While both of these studies found a negative association between atherosclerosis and sirtuin expression, Kilic et al.^30^ found that serum sirtuin 1 levels were higher in stable CAD patients than in the control patients.^30^

By taking all these data together, the causality between sirtuins and human atherosclerosis needs more investigation. Additionally, we firstly investigated the temporal change of serum sirtuin 1, 3 and 6 levels in the AMI course and did not found any significant change between admission, first and second day of AMI. Another issue needs to be addressed is the association between inflammation and sirtuin levels. In our study, serum sirtuin 1 and 3 levels were not correlated and serum sirtuin 6 levels were positively correlated with serum CRP levels, a widely accepted inflammatory marker. As we mentioned above, experimental studies suggested that sirtuins have anti-inflammatory effects and this is hypothesized as one of the mechanisms for atheroprotection.^31^ We expected to find a negative association between sirtuins and CRP, but the results were not concordant with our hypothesis. Although sirtuin 6 has an anti-inflammatory feature, a possible positive regulatory role for SIRT6 in the induction of pro-inflammatory cytokine expression is evident both in innate and adaptive immune cells. Thus our positive correlation finding between sirtuin 6 and CRP can be interpreted in this context.^32^

Our findings regarding the association between sirtuins and the parameters reflecting infarction size like peak troponin, ejection fraction and pro BNP levels were also remarkable. Opening the occluded artery and restoring the blood flow to the myocardium is the mainstay of the AMI management. However, it was suggested that restoration of blood flow may account for further myocardial damage and it is termed as reperfusion injury.^33^ Microvascular obstruction, myocyte hypercontracture and contraction band necrosis, free radical generation and inflammatory cell accumulation are the proposed mechanisms for reperfusion injury.^34^

Ischemic preconditioning, which reduces reperfusion injury can be defined as transient, sublethal ischemic episodes rendering the myocardium more resistant to a sustained, lethal ischemic period. Since first described by Murry et al.,^35^ ischemic preconditioning has become the most relevant entity against reperfusion injury. Ischemic preconditioning requires complex intracellular molecular interactions and the molecular mechanism is still unclear.^36^ It is suggested that some sirtuin types reduces ischemia reperfusion injury. In an experimental study, inhibition of sirtuin 1 resulted in reduction of bakuchiol induced cardioprotection in rat hearts.^37^ Opening of the mitochondrial permeability transposition pores are crucial in the pathophysiology of ischemia reperfusion injury. Sirtuin 3 deacetylates the regulatory component of the mitochondrial permeability transposition pore and sirtuin 3 deficient mice myocytes exhibited an increase in mitochondrial swelling due to increase in the number of opened mitochondrial permeability transposition pores.^38^ In this study, we investigated whether there was any association between serum sirtuin levels and infarct size markers. There was no correlation between serum sirtuin levels and peak troponin levels, LVEF and pro BNP levels in AMI patients. These findings reflect that serum sirtuin 1, 3 and 6 levels were not associated with the infarct size. Pre infarction angina pectoris can be used as the surrogate marker of ischemic preconditioning in daily practice and is associated with reduced peak troponin levels in AMI patients.^39^ Although the number of subjects were low to make a firm conclusion, no difference was evident about serum sirtuin 1, 3 and 6 levels in the patients with and without pre infarction angina.

Finally, we should mention about the association between serum sirtuin 3 levels and GRACE score, and between reperfusion duration and serum sirtuin 1/6 levels. GRACE score is a well-known and validated risk score for morbidity and mortality in AMI patients.^16^ Although we could not find any correlation between serum sirtuin 3 levels and prognostic markers like peak troponin, pro-BNP and LVEF, the negative correlation between serum sirtuin 3 levels and GRACE score may be an inspiration for further studies to investigate the role of serum sirtuin 3 levels for the risk assessment in AMI patients. We found a positive correlation between serum sirtuin 1/sirtuin 6 levels and reperfusion duration. The duration between symptom onset and reperfusion was not a marker of mortality in STEMI patients.^40^ Although correlated with reperfusion duration positively, sirtuin 1 and sirtuin 6 levels were not correlated with prognostic markers in AMI patients.

There are some limitations of our study. The small sample size seems the most important limitation of the study. The other important limitation is the methodological differences with previous studies. In previous studies, sirtuin analysis was performed with specimens from atherosclerotic plaques or peripheral mononuclear cells. Sirtuin mRNA levels and protein expression levels were evaluated in those studies. In our study, we directly measured serum sirtuin levels. Although our methodology was not the same with other studies, we firstly investigated serum sirtuin levels in our particular study population. This can be accepted as the originality of our study.

## Conclusion

Serum sirtuin 1, sirtuin 3 and sirtuin 6 levels did not significantly differ between AMI patients and patients with normal coronary arteries. No temporal change was found in the serum levels of these sirtuins in the AMI course and there was no correlation between the serum levels of these sirtuins and the parameters reflecting myocardial infarct size like peak troponin level, LVEF and pro-BNP. We detected a negative correlation between sirtuin 3 and GRACE score, as a secondary finding. Cardioprotective role of serum sirtuin 1, 3 and 6 needs more investigation in AMI patients.
